# Serum Oxidative Stress-Induced Repression of Nrf2 and GSH Depletion: A Mechanism Potentially Involved in Endothelial Dysfunction of Young Smokers

**DOI:** 10.1371/journal.pone.0030291

**Published:** 2012-01-17

**Authors:** Anna Fratta Pasini, Anna Albiero, Chiara Stranieri, Mattia Cominacini, Andrea Pasini, Chiara Mozzini, Paola Vallerio, Luciano Cominacini, Ulisse Garbin

**Affiliations:** Department of Medicine, Section of Internal Medicine D, University of Verona, Verona, Italy; University of Udine, Italy

## Abstract

**Background:**

Although oxidative stress plays a major role in endothelial dysfunction (ED), the role of glutathione (GSH), of nuclear erythroid-related factor 2 (Nrf2) and of related antioxidant genes (ARE) are yet unknown. In this study we combined an *in vivo* with an *in vitro* model to assess whether cigarette smoking affects flow-mediated vasodilation (FMD), GSH concentrations and the Nrf2/ARE pathway in human umbilical vein endothelial cells (HUVECs).

**Methods and Results:**

52 healthy subjects (26 non-smokers and 26 heavy smokers) were enrolled in this study. In smokers we demonstrated increased oxidative stress, i.e., reduced concentrations of GSH and increased concentrations of oxidation products of the phospholipid 1-palmitoyl-2-arachidonyl-sn-glycero-3-phosphorylcholine (oxPAPC) in serum and in peripheral blood mononuclear cells (PBMC), used as *in vivo* surrogates of endothelial cells. Moreover we showed impairment of FMD in smokers and a positive correlation with the concentration of GSH in PBMC of all subjects. In HUVECs exposed to smokers' serum but not to non-smokers' serum we found that oxidative stress increased, whereas nitric oxide and GSH concentrations decreased; interestingly the expression of Nrf2, of heme oxygenase-1 (HO-1) and of glutamate-cysteine ligase catalytic (GCLC) subunit, the rate-limiting step of synthesis of GSH, was decreased. To test the hypothesis that the increased oxidative stress in smokers may have a causal role in the repression of Nrf2/ARE pathway, we exposed HUVECs to increasing concentrations of oxPAPC and found that at the highest concentration (similar to that found in smokers' serum) the expression of Nrf2/ARE pathway was reduced. The knockdown of Nrf2 was associated to a significant reduction of HO-1 and GCLC expression induced by oxPAPC in ECs.

**Conclusions:**

In young smokers with ED a novel further consequence of increased oxidative stress is a repression of Nrf2/ARE pathway leading to GSH depletion.

## Introduction

Increasing data support the hypothesis that oxidative stress provides an important pathophysiological link between cigarette smoking and atherosclerosis [1], [2]. Both the gas and tar phases of cigarette smoke (CS) deliver a high concentration of oxidizing chemicals, including reactive oxygen species (ROS), nitric oxide (NO), peroxynitrite and free radicals [2], [3] that can get into the bloodstream and cause macromolecular damage in the vascular cells [4].

The phospholipid 1-palmitoyl-2-arachidonyl-sn-glycero-3-phosphorylcholine (PAPC) is a major component of cell membranes and lipoproteins. Oxidation products of PAPC (oxPAPC) are found in atherosclerotic lesions, in cells during inflammation, in membranes of apoptotic cells, as well as in oxidized low density lipoproteins [5]. OxPAPC triggers inflammatory responses *in vitro* and *in vivo* via increased expression of inflammatory genes and the activation of monocyte binding to endothelial cells [6]. Among the different oxPAPC 1-palmitoyl-2-(5,6-epoxyisoprostane E2)-sn-glycero-3-phosphocholine (PEIPC), 1-palmitoyl-2-(5-oxovaleroyl)-sn-glycero-3-phosphorylcholine (POVPC) and 1-palmitoyl-2-glutaroyl-sn-glycero-3-phosphorylcholine (PGPC) have been previously identified as the most bioactive components [7].

Cigarette smoking is also associated with a depletion of circulating endogenous antioxidants [1], [2]. The tripeptide glutathione (GSH) plays a key role in maintaining intracellular reduction–oxidation (redox) balance and in establishing the mechanisms of cellular defences augmented by oxidative stress [8], [9]. The rate-limiting step of the *de novo* synthesis of GSH is catalysed by glutamate-cysteine ligase, a heterodimer composed of a catalytic heavy chain (GCLC) and a modifier (or regulatory) light chain [9]. The transcription of human GCLC is mainly regulated by nuclear erythroid-related factor 2 (Nrf2), a redox-sensitive transcription factor that plays a major role in antioxidant response element (ARE)-mediated induction of antioxidant enzymes [10]. Under basal conditions, Nrf2-dependent transcription is repressed by its negative regulator Keap1. When cells are exposed to oxidative stress or electrophiles, Nrf2 accumulates in the nucleus and drives the expression of its target genes [10]. Among the spectrum of antioxidant genes controlled by Nrf2 heme-oxygenase-1 (HO-1) [11] and GCLC are of particular interest, especially in CS-induced oxidative stress [12].

Endothelial dysfunction is well established as one of the primary events in the pathogenesis of atherosclerosis [13] and a predictor of cardiovascular (CV) disease [14]. Previous studies have shown that long-term cigarette smoking produces impairment of flow-mediated dilation (FMD) [15]–[17]. Although decreased NO bioavailability and increased generation of ROS play a critical role in CS-related endothelial dysfunction [15], [18]–[19] there is recent evidence showing that GSH is important in the regulation of endothelial dysfunction in smokers [20]. However the precise mechanisms and, in particular, the role of the protective Nrf2/ARE signalling pathway are not yet known. We have recently demonstrated an increased formation of oxPAPC and a negative regulation of Nrf2/ARE pathway in peripheral blood mononuclear cells (PBMC) of young healthy heavy smokers [21]. Therefore, the aim of this study was to combine an *in vivo* model with an *in vitro* model to assess whether cigarette smoking affects FMD, GSH concentrations and the Nrf2/ARE pathway in endothelial cells. Accordingly, we first evaluated FMD together with GSH and oxPAPC in serum and in PBMC of young smokers compared to non-smokers. Then serum from the same individuals and increasing concentrations of oxPAPC were used to assess the effect of smoking on intracellular GSH, ROS and NO concentration, and on the expression of Nrf2/ARE pathway in endothelial cells.

## Methods

### Ethics statement

The study was approved by the Ethical Committee of University of Verona and all participants provided written consent prior to commencing the study. All clinical investigations were conducted according to the principles expressed in the Declaration of Helsinki.

### Study Population

Fifty-two (26 males and 26 females) healthy subjects, 20–35 years of age (mean 28.2±5 years) were enrolled in the study. None of the participants had a history of hypertension, hypercholesterolemia or diabetes mellitus, drank more than five units of alcohol per week, were following a weight-reducing diet or exercised more than 3×30 min aerobic exercise per week. Furthermore, none of the participants took antioxidants, anti-inflammatory or cardiovascular medications during or for six months prior to commencing the study. Of the 52 subjects, 26 were active cigarette smokers with a mean cigarette number of 22.7±3.7/day, a mean duration of smoking of 12.6±2.2 years, a mean cumulative cigarette consumption of 14.8±4.3 pack/years and were thus assigned to the smoking group. A pack year is defined as twenty cigarettes smoked every day for one year. Smokers were asked to refrain from smoking for at least 8 hours. The remaining 26 subjects did not have a history of cigarette smoking and were assigned to the non-smoking group.

### Evaluation of FMD

Endothelial function was assessed using a high-resolution B-mode ultrasound (Envisor, Philips) equipped with a 7.5-to 12-MHz linear array transducer to measure FMD of the brachial artery (BA) in response to hyperaemia, which is currently the most frequently used non-invasive surrogate of endothelial function [22], [23]. A B-mode scan of the right BA was obtained in longitudinal section between 5 and 10 cm above the elbow; the transducer was held at the same point throughout the scan by a stereotactic clamp. After 1 min of acquisition to measure basal diameter, a pneumatic cuff below the antecubital fossa (lower arm occlusion) was inflated 50 mmHg above the systolic pressure for 5 min. Following deflation, the BA was imaged continuously for 3 min (endothelium-dependent dilation). A repeat baseline scan was obtained after a 15-min rest. Endothelium-independent dilation was obtained by the administration of sublingual glyceryl trinitrate (GTN, 25 µg). We chose this small dose because it gives an equivalent dilation to FMD, allows assessment of smooth muscle function and reduces the concerns regarding potential side-effects from large doses of GTN [23]. FMD and GTN-induced dilation were defined as the maximal percent increment of the diameter value with respect to the baseline, measured by a recently developed system for real-time measurement of BA diameter (FMD Studio, Institute of Clinical Physiology, CNR Pisa, Italy) [24]. All exams were performed by the same operator blind to the characteristics of the subjects. Each female subject was studied in menstrual phase, since FMD in male subjects is comparable to that in female subjects only in this phase [25].

### Blood samples and PBMC isolation

Venous blood samples were obtained from each subject after 12 hours fasting and drawn into pyrogen-free blood collection tubes. Multiple aliquots of serum were placed into sterile 1-ml screw-capped polypropylene vials with phenolic antioxidant 2,6-Di-tert-butyl-4-methylphenol 10 mmol/L (Sigma) added to inhibit lipid peroxidation and stored at −80°C. The samples were frozen and thawed only once. PBMC were isolated as previously described [26]. Briefly, whole blood was layered onto a sterile aqueous medium containing ficoll and sodium diatrizoate at a predetermined density of 1.007 g/mL at 25°C. Gentle centrifugation at room temperature resulted in the separation of PBMC at the blood/ficoll interface, with the other white and red blood cells passing through the interface. Total cholesterol, high density lipoprotein (HDL) cholesterol, low density lipoprotein (LDL) cholesterol, triglycerides and glucose were measured with standard methods.

### Evaluation of oxPAPC in serum and in PBMC from non-smokers and smokers

OxPAPC in serum and in PBMC from non-smokers and smokers were measured on an Agilent mass spectrometer equipped with an electrospray source as previously described [21], [27]. The following different oxPAPC: PEIPC, POVPC and PGPC were taken into consideration. Flow injection experiments were performed by an HPLC system (HP1100; Agilent Technologies). Quantification of the peak areas was performed by single ion monitoring in the elution time range of 10–20 min using appropriate software. Authentic PAPC, POVPC (594.4 m/z) and PGPC (610.2 m/z) were obtained from Avanti Polar Lipids, Inc. (Alabaster, AL). PEIPC (828.5 m/z) was prepared and analyzed in our laboratory as previously described [28].

#### Cell culture

Human umbilical vein endothelial cells (HUVECs) from single donor were isolated, cultured as previously described [29] and used at passages 2–4. Early apoptosis and cells viability were determined by using the AnnexinV-FITC Kit (Bender MedSystem GmbH, Vienna, Austria) and 7-amino-actinomycin D (7-AAD) (BD Biosciences) [30] by flow cytometry; cell proliferation was monitored by using a commercial kit (Chemicon). Based on previous observations [15], [31], HUVECs were incubated without and with increasing amounts (10, 30 and 50%) of serum derived from non-smokers and smokers at different time points (0, 6 and 12 hours). Under these experimental conditions, no change of Annexin V was observed compared with untreated cells (data not shown) and no significant differences were observed in viability and proliferation (data not shown) among HUVECs non treated or treated with serum. This allowed us to conclude that any differences were not attributable to a toxic effect on HUVECs. Endotoxin contamination of cell cultures involving the use of serum was routinely excluded with the chromogenic Limulus amoebocyte lysate assay (Sigma). HUVECs were also incubated with the corresponding amounts (10, 30 and 50%) of lipoprotein-depleted serum (LPDS) derived from smokers, in which all the lipoproteins were taken away by ultracentrifugation at a density >1.21 g/mL, as previously described [32].

In these experiments HUVECs were incubated with serum and LPDS for 12 hours at 37°C because this time point allowed us to evaluate GSH, ROS, NO and Nr2/ARE pathway expression without affecting cell viability and proliferation.

### Preparation of oxPAPC

PAPC was oxidized by air exposure for 48 hours and the composition of oxPAPC was analyzed by ESI-MS Agilent as previously described (28). 75 mg of oxPAPC yelded about 0.6–0.7 µg/mL (1.01–1.17 µmol/L) of POVPC, about 0.38–0.45 µg/mL (0.62–0.74 µmol/L of PGPC) and about 1.0–1.1 µg/mL (1.20–1.32 µmol/L) of PEIPC. The lipids were extracted with chloroform and resuspended in methanol before the addition to the HUVECs. For the experiments HUVECs were incubated without and with increasing concentrations (25–150 µg/mL) of oxPAPC in M-199 containing 0.2% foetal calf serum (FCS) for 6 hours at 37°C. The concentration of oxPAPC (150 µg/mL) contained concentrations of POVPC, PGPC and PEIPC similar to those found in serum of smokers.

### GSH measurement in serum, PBMC and in HUVECs

The detailed procedures for the measurement of cellular and serum GSH have been previously described [33]. Samples were directly collected into specially prepared tubes containing the preservative 2,6-Di-tert-butyl-4-methylphenol (10 mmol/L) to reduce auto-oxidation and frozen at −80°C. Samples were analysed using high performance liquid chromatography with fluorescence detection of 7-fluorobenzo-2-oxa-1,3-diazol-4-sulfonic acid at excitation 385 nm and emission 515 nm.

### Effect of increasing amounts of serum derived from non-smokers and smokers, of LPDS derived from smokers and of increasing concentrations of oxPAPC on ROS measurement

Intracellular ROS levels were measured as described [29], by following the oxidation of 2′,7′-dichlorofluorescin diacetate (DCFH-DA) (Molecular Probes, Eugene, USA) by flow cytometry (Coulter Corporation, Hialeah, Florida). Confluent HUVECs in 24-well tissue culture plates were incubated without (control) and with increasing amounts (10–50%) of serum (total volume 300 µL) derived from non-smokers and smokers and with LPDS derived from smokers; the supernatants were removed after 12 hours, cells washed and 10 µmol/L DCFH-DA was added to M-199 containing 10% FCS for 20 min at 37°C. To evaluate the effect of oxPAPC on intracellular ROS concentrations, some experiments were performed by adding increasing concentrations (25–150 µg/mL) of oxPAPC.

### Effect of increasing amounts of serum derived from non-smokers and smokers, of LPDS derived from smokers and of increasing concentrations of oxPAPC on NO production in HUVECs

NO production was monitored by following levels of nitrite in the supernatants of HUVECs by a fluorimetric assay as previously described [31], [34].

Basal NO production in culture was measured by incubating HUVECs without (control) and with increasing amounts (10–50%) of serum (total volume 300 µL) derived from non-smokers and smokers, with LPDS derived from smokers and with increasing concentrations (25–150 µg/mL) of oxPAPC in 24-well tissue culture plates. After 12 hours, basal NO production in the supernatant was determined. To measure stimulated NO production in culture, the supernatants were removed after 12 hours, the cells were washed and fresh medium was added to each well followed by stimulation with bradykinin (100 nmol/L), for 10 min at 37°C in the presence of 5 mmol/L arginine and 3 µmol/L tetrahydrobiopterin.

### Effect of serum derived from non-smokers, of LPDS derived from smokers, and of increasing amounts of serum derived from smokers and of increasing concentrations oxPAPC on Nrf2/ARE pathway expression in HUVECs

HUVECs were incubated for 12 hours at 37°C with 50% of serum derived from non-smokers, 50% of LPDS derived from smokers and with increasing amounts (10–50%) of serum derived from smokers. HUVECs were also incubated for 6 hours in M-199 containing 0.2% FCS with increasing concentrations (25–150 µg/mL) of oxPAPC.

### Real time RT-PCR quantification of RNA Nrf2, GCLC and HO-1 expression

#### Quantitative Real-Time PCR

Total RNA was extracted, and real-time RT-PCR was conducted as previously described [21], [26]. In brief, RNA was extracted from HUVECs with an RNeasy Mini Kit (Qiagen) and was reverse transcribed using an IScript cDNA Synthesis Kit (Bio-Rad, Hercules, CA). The relative expression levels of mRNA encoding Nrf2, GCLC, HO-1 and β-actin were measured by iCycler (Bio-Rad, Hercules, CA), using IQSYBR Green PCR SuperMix (Bio-Rad, Hercules, CA) and 300 nM of each primer pair. Primers were designed by Beacon Design 4.0 software (PREMIER Biosoft International, Palo Alto, CA, USA) and synthesised by MWG Biotech AG (Ebersberg, Germany): Nrf2, sense 5′-TTCAGCCAGCCCAGCACATC-3′ and antisense 5′-CGTAGCCGAAGAAACCTCATTGTC-3′; GCLC, sense 5′ CGAATTCGCCAAGAATGAGGAGATCC-3′, antisense 5′- CGAATTCGAAAGCGACGGCTGTACC-3′; HO-1, sense 5′-GGTGACCCGAGACGGCTTC-3′ and antisense 5′-AGACTGGGC TCTCCTTGTTGC- 3′; β-actin, sense 5′- ATCAAGATCATTGCTCCTCCTG-3 and antisense 5′- GCAACTAAGTCATAGTCCGCC-3′. Expression levels were normalised to the level of β-actin.

### Western blotting analysis

Western blot analysis for GCLC and HO-1 protein expression in HUVECs was performed as previously described [21], [26]. Nuclear extract for Nrf2 was obtained by utilizing a nuclear extract kit from Active Motif (Rixensart, Belgium).

### siRNA-mediated knockdown of Nrf2

HUVECs were transfected with siRNA against Nrf2 siRNA (5′ GUUUUUCCAGCUCAUACUCUUTT-3′), or negative control (Invitrogen) as previously described [35] for 24 hours and then treated with 75 µg/mL oxPAPC for 6 hours for the measurement of the expression levels of GCLC and HO-1.

### Statistical analysis

Data are expressed as mean±SD values if normally distributed. Differences between two groups were analysed by a two-tailed unpaired Student's t-test. Statistical comparison among three groups was performed by one-way ANOVA. The relationship between variables was assessed by linear regression. A probability value (P) of 0.05 was considered to be statistically significant. All data were analysed with StatView (SaS).

## Results

No significant differences existed between the two groups of non-smokers and smokers with respect to age (27.2±4.4 versus 29.3±5.6 years, respectively), gender (male/female: 14/12 versus 12/14, respectively), waist circumference (74.2±11.8 versus 73.2±10.4 cm, respectively), body mass index (22.9±2.7 versus 21.4±3.0, respectively), systolic (114.2±8.9 versus 117.6±9.8 mmHg, respectively) and diastolic (71.3±8.3 versus 73.9±7.5 mmHg, respectively) blood pressure, heart rate (69.1±8.8 versus 73.5±8.1 beat/min, respectively), total cholesterol (179.7±33 versus 188.4±29.3 mg/dl, respectively), LDL cholesterol (109.5±26.1 versus 124.3±19.7 mg/dl, respectively), HDL cholesterol (44.5±10.2 versus 46.4±11.9 mg/dl, respectively), total cholesterol/HDL (4.07±0.3 versus 4.04±0.5) and plasma glucose (73.4±8.9 versus 74.2±8.5 mg/dl, respectively). In contrast, smokers had significantly higher concentrations of oxPAPC both in serum (POVPC 1.02±0.31 µmol/L, PGPC 2.80±0.39 µmol/L and PEIPC 3.74±0.52 µmol/L, P<0.01) and in PBMC (POVPC 2.2±0.23 ng/mg PAPC, PGPC 7.14±0.91 ng/mg PAPC, PEIPC 4.85±0.47 ng/mg PAPC, P<0.01) compared to non-smokers (serum: POVPC 0.32±0.14 µmol/L, PGPC 1.06±0.29 µmol/L, PEIPC 0.95±0.22 µmol/L; PBMC: POVPC 0.61±0.09 ng/mg PAPC, PGPC 2.13±0.57 ng/mg PAPC, and PEIPC 1.91±0.51 ng/mg PAPC, P<0.01). Furthermore, the concentrations of GSH were significantly lower in smokers than in non-smokers both in serum (2.6±0.43 µmol/L versus 5.7±0.78 µmol/L, respectively, P<0.01) and in PBMC (1.42±0.23 ng/mg cell protein versus 2.93±0.32 ng/mg cell protein, respectively, P<0.01).

### Endothelial function

**Figure 1:**
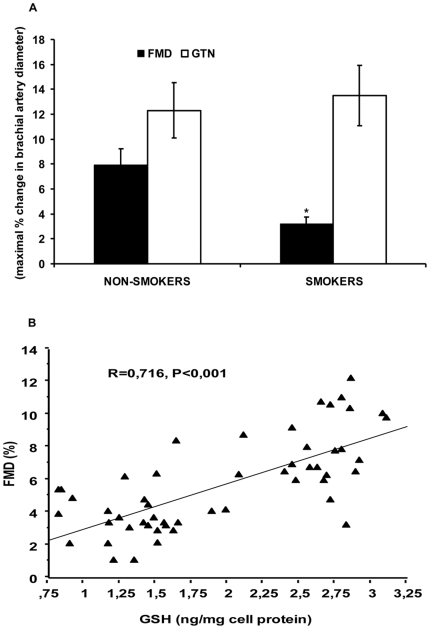
Endothelial function and correlation between flow-mediated vasodilation (FMD) and concentrations of GSH in peripheral blood mononuclear cells (PBMC) of non-smokers and smokers. FMD and glyceryl trinitrate (GTN)-induced vasodilation in non-smokers and smokers (A); correlation between FMD and intracellular concentrations of GSH in PBMC of non-smokers and smokers (B). Data are presented as mean±SD; FMD and GTN are expressed as maximal percentage change in brachial artery dilation. *P<0.001 versus non-smokers.

FMD was significantly impaired in smokers compared to non-smokers (3.2±1.6 versus 7.9±2.8%, respectively, P<0.001); on the contrary, GTN-induced dilation (12.3±4.1 versus 13.5±3.8%, respectively) was similar in both groups, indicating that our subjects were apparently free of smooth muscle cell disorders. These data are shown in Figure 1a. Moreover, BA diameters (3.52±0.4 versus 3.83±0.5 mm, respectively) were not different between non-smokers and smokers. Interestingly, we found a significant positive correlation between the concentrations of GSH in PBMC and FMD in all subjects (r = 0.716, P<0.001), as shown in Figure 1b.

### Effect of serum derived from non-smokers and smokers and of LPDS derived from smokers on intracellular GSH, ROS and NO concentration, and on Nrf2/ARE pathway (mRNA and protein) expression in HUVECs

**Figure 2:**
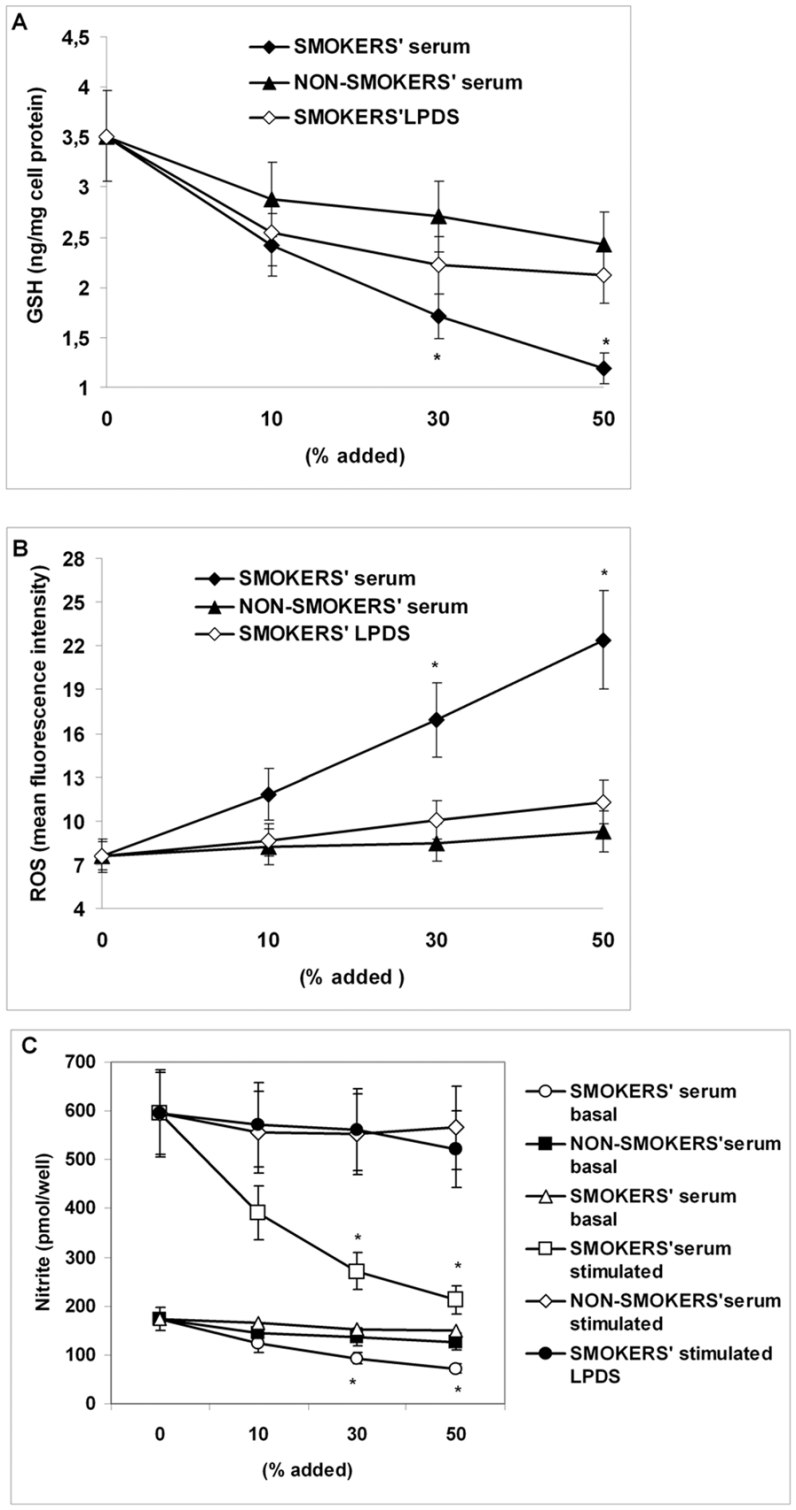
Effect of serum derived from non-smokers and smokers and of lipoprotein-depleted serum (LPDS) derived from smokers on intracellular GSH concentration, on intracellular reactive oxygen species (ROS) and on nitric oxide (NO) formation in human umbilical vein endothelial cells (HUVECs). Confluent HUVECs were incubated without and with 10, 30 and 50% serum derived from non-smokers and smokers and with the corresponding LPDS derived from smokers for 12 hours. Figure shows intracellular GSH concentration (A), intracellular ROS (B) and cumulative basal and bradykinin-stimulated NO production, evaluated by measuring levels of nitrite in the supernatants (C). Data are presented as mean±SD of measurements performed in triplicate in four different occasions; *P<0.01 versus control (no addition of serum derived from the subjects) non-smokers' serum and smokers' LPDS.

Incubation of HUVECs with increasing amounts of serum derived from smokers for 12 hours resulted in a dose-dependent significant decrease (P<0.01, at 30 and 50%) of GSH concentrations compared to serum derived from non-smokers (Figure 2a). Moreover serum derived from smokers induced a dose-dependent increase (P<0.01 at 30 and 50%) of intracellular ROS formation (Figure 2b), whereas serum from non-smokers did not. The cumulative production of NO as evaluated by measuring levels of nitrite in the supernatant of cell culture was significantly increased after stimulation of HUVECs with bradykinin (Figure 2c). The levels of nitrite in the supernatant of basal and bradykinin-stimulated endothelial cells dose-dependently dropped (P<0.01 at 30 and 50%) after preincubation of HUVECs with serum derived from smokers; on the contrary, serum derived from non-smokers did not reduce nitrite concentration in the supernatants of HUVECs (Figure 2c).

Since the majority of circulating oxPAPC is carried by lipoproteins [6], we used LPDS to eliminate its effect from smokers' serum. The incubation of HUVECs with smokers' LPDS almost completely abolished the increase in ROS and the decrease in GSH and in nitrite induced by smokers' serum (Figure 2 a–c).

**Figure 3:**
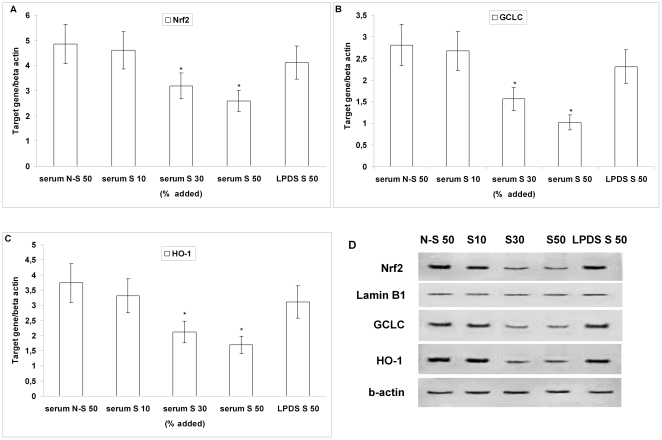
Effect of serum derived from non-smokers and smokers and of lipoprotein-depleted serum (LPDS) derived from smokers on Nrf2, GCLC and HO-1 expression in human umbilical vein endothelial cells (HUVECs). Confluent HUVECs were incubated without and with increasing amounts (10, 30 and 50%) of serum derived from smokers (serum S 10, serum S 30, serum S 50), with 50% serum derived from non-smokers (serum N–S 50) and with 50% LPDS derived from smokers (LPDS S 50) for 12 hours. mRNA for Nrf2, GCLC and HO-1 (A–C) was analysed by quantitative Real-Time PCR. Normalised gene expression levels were given as the ratio between the mean value for the target gene and that for beta-actin in each sample. Results are reported as the mean±SD of measurements performed in triplicate. *P<0.01 versus non-smokers and LPDS. (D) shows a representative Western blot analysis of three independent experiments for nuclear Nrf2, for GCLC and HO-1.

To determine whether intracellular GSH depletion may be due to its reduced synthesis and based on these results, we then evaluated the effect of increasing amounts (10–50%) of serum derived from smokers, of serum derived from non-smokers and of LPDS (50%) on Nrf2 and GCLC expression. Our results show that both Nrf2 (Figure 3a) and GCLC (Figure 3b) mRNA and protein expression (Figure 3d) was dose-dependently decreased (P<0.01 at 30 and 50%) in HUVECs incubated with serum derived from smokers, compared to HUVECs incubated with serum of non-smokers and with LPDS derived from smokers. To further examine the consequences of nuclear levels of Nrf2 we also examined the expression of another Nrf2-regulated antioxidant gene, HO-1. As shown in Figure 3, serum derived from smokers also dose-dependently reduced (P<0.01 at 30 and 50%) both mRNA (c) and protein expression (d) of HO-1, compared to serum derived from non-smokers and with LPDS derived from smokers.

### Effect of increasing concentrations of oxPAPC on intracellular ROS, NO and GSH concentrations, and on Nrf2, GCLC and HO-1 (mRNA and protein) expression in HUVECs

**Figure 4:**
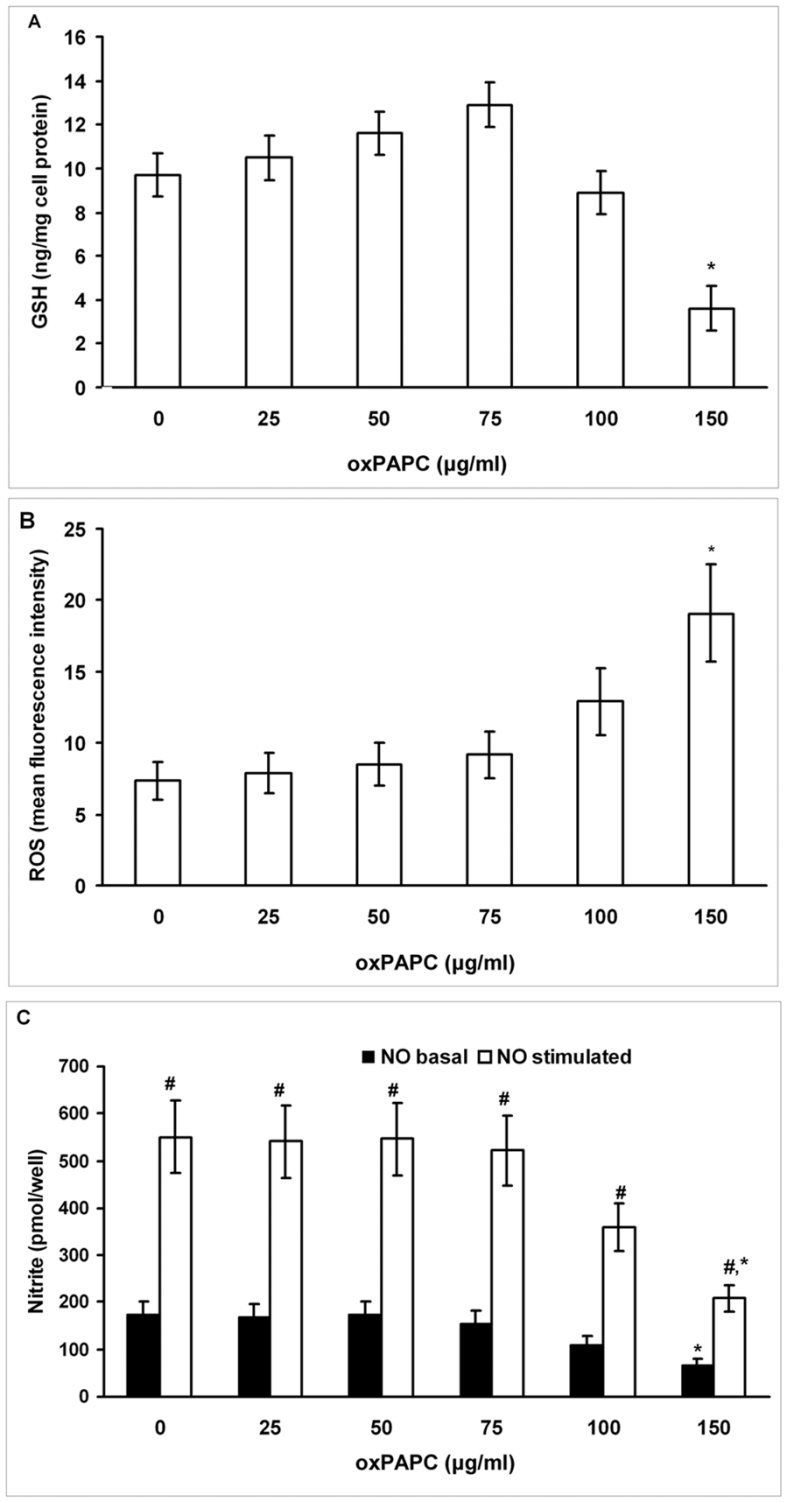
Effect of increasing concentrations of oxPAPC on intracellular GSH concentration, on intracellular reactive oxygen species (ROS), and on nitric oxide (NO) production in human umbilical vein endothelial cells (HUVECs). Confluent HUVECs were incubated without and with increasing concentrations (25–150 µg/mL) of oxPAPC for 6 hours. Figure shows intracellular GSH concentration (A), intracellular ROS (B) and cumulative basal and bradykinin-stimulated NO production, evaluated by measuring levels of nitrite in the supernatants (C). Data are presented as mean±SD of measurements performed in triplicate in four different occasions. *P<0.01 versus oxPAPC 0 e 75 µg/mL. # P<0.01 versus basal NO.

To test the hypothesis that the increased oxidative stress in smokers may have a causal role in the repression of Nrf2/ARE pathway which in turn may reduce GSH concentration, we exposed HUVECs to increasing concentrations of oxPAPC (from 25 to 150 µg/mL) for 6 hours. Figure 4a shows a progressive slight increase of intracellular GSH concentration at the lowest concentrations (from 25 to 75 µg/mL), whereas at the highest concentration, GSH was significantly decreased (P<0.01). The incubation of HUVECs with increasing concentrations of oxPAPC induced a dose-dependent increase (P<0.01 at the highest concentration) in intracellular ROS formation (Figure 4b); on the contrary, a dose-dependent decrease (P<0.01 at the highest concentration) in basal and bradykinin-induced intracellular NO concentration was observed (Figure 4c).

**Figure 5:**
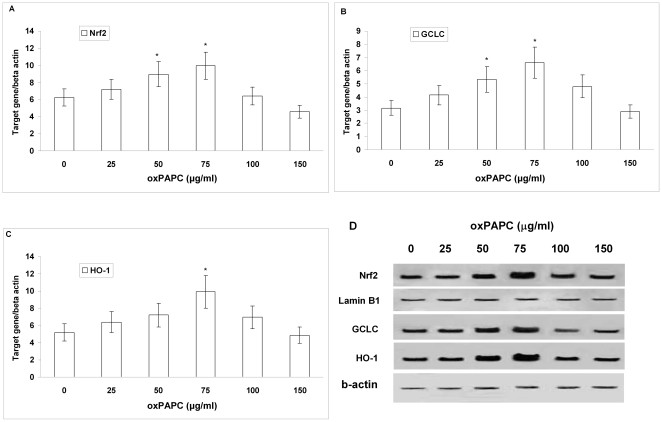
Effect of increasing concentrations of oxPAPC on Nrf2, GCLC and HO-1 expression in human umbilical vein endothelial cells. mRNA (A–C) was analysed by quantitative Real-Time PCR. Normalised gene expression levels were given as the ratio between the mean value for the target gene and that for beta-actin in each sample. Results are presented as the mean±SD of measurements performed in triplicate.*P<0.01 versus oxPAPC 0 and 150 µg/mL. Figure (D) shows a representative Western blot analysis of three independent experiments for nuclear Nrf2, GCLC and HO-1.

Our results also show that Nrf2, GCLC and HO-1 (mRNA and protein) expression was dose-dependently increased in HUVECs incubated with the lowest concentrations of oxPAPC (from 25 to 75 µg/mL, P<0.01), while Nrf2, GCLC and HO-1 (mRNA and protein) expression was repressed in HUVECs incubated with the highest concentration of oxPAPC, as shown in Figure 5 (a–d).

### Nrf2 is required for oxPAPC-stimulated GCLC and HO-1 expression

**Figure 6:**
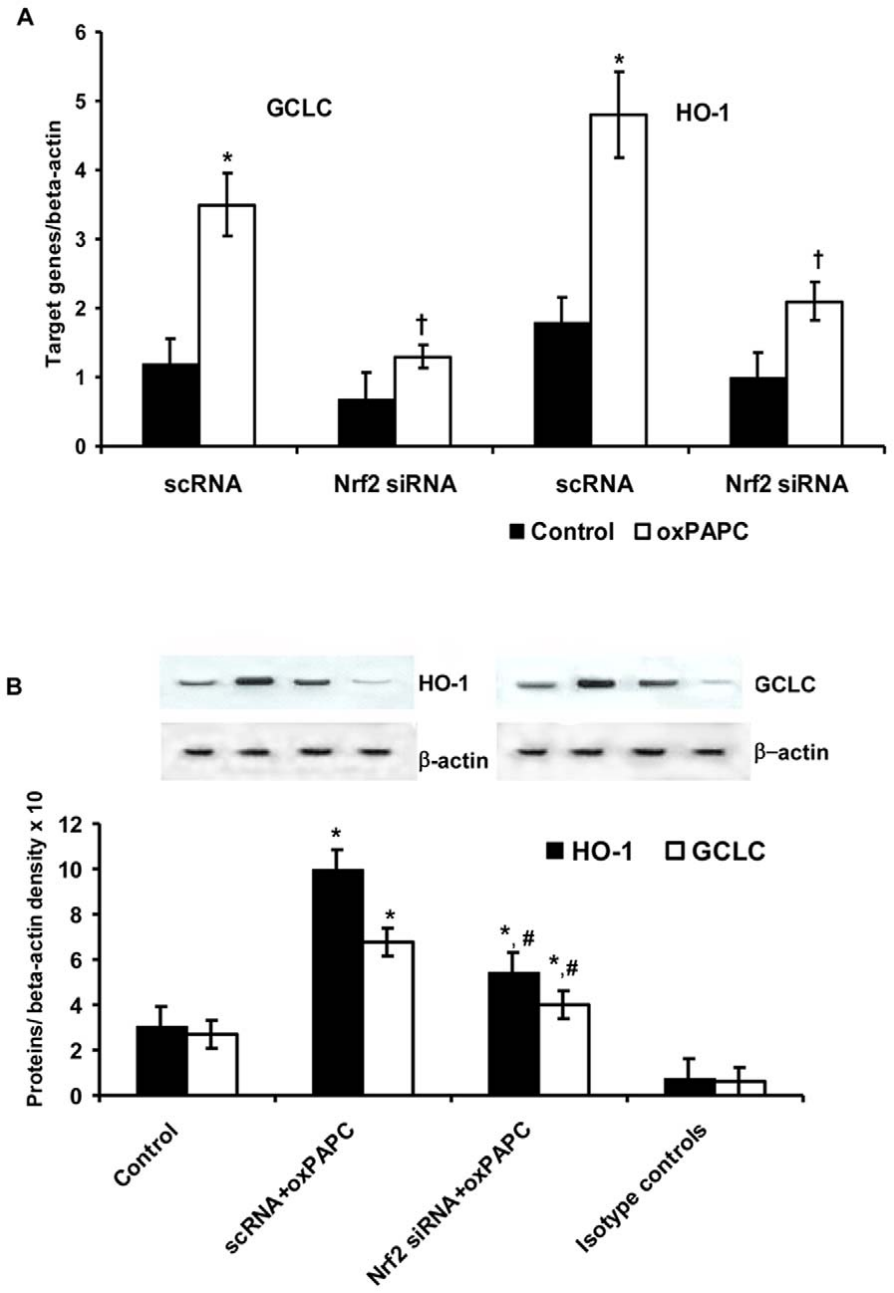
Effect of small interfering (si) and scrambler (sc) RNA against Nrf2 on oxPAPC-dependent expression of GCLC and HO-1 in human umbilical vein endothelial cells. mRNA (a) was analyzed by quantitative Real-Time PCR. Normalized gene expression levels were given as the ratio between the mean value for the target gene and that for the beta-actin in each sample. Figure shows a representative Western blot analysis for GCLC and HO-1 and the average quantification obtained by densitometric analysis of all the samples (b). Data on Western blot analysis are expressed as the density ratio of target to control (beta-actin) in arbitrary units ×10. Results are the mean±SD of measurements performed in triplicate in four different occasions. *P<0.01 versus control; †P<0.01 versus scRNA; #P<0.01 versus oxPAPC+scRNA.

To determine whether Nrf2 is required for oxPAPC-stimulated GCLC and HO-1 expression, we used siRNA to knockdown the expression of Nrf2. Nrf2 siRNA significantly reduced Nrf2 mRNA expression (data not shown). Figure 6 (a and b) shows that the knockdown of Nrf2 was associated to a significant reduction (P<0.01) of GCLC and HO-1 (mRNA and protein) expression induced by oxPAPC in HUVECs.

## Discussion

The core findings of our study performed in young, otherwise healthy subjects are the following: 1) FMD, which was significantly impaired in the smoking group, positively correlated with the concentrations of GSH in PBMC of all subjects; 2) PBMC obtained from young smokers, used as *in vivo* surrogates of endothelial cells, showed lower concentrations of GSH and higher concentrations of oxPAPC than non-smokers. We explored the possible mechanism by which endothelial GSH depletion affects endothelial function and found that in HUVECs exposed to the highest amount of smokers' serum or when cells where incubated with the highest concentration of oxPAPC: a) GSH concentrations were significantly reduced, b) ROS formation was significantly increased, whereas NO concentration was significantly decreased, c) the expression of the protective Nrf2 and of downstream genes was significantly reduced. Although the effects of smokers' serum on HUVECs may be dependent from different factors than oxPAPC, the fact that smokers' LPDS did not reduce GSH and did not repress Nrf2/ARE pathway expression strongly suggests that oxPAPC may play a key role in smokers' serum-dependent effects in HUVECs.

Taken together our results suggest that, in young heavy smokers with endothelial dysfunction, oxidative stress induced by cigarette smoking results in a repression of the Nrf2/ARE pathway leading to intracellular GSH deficiency.

Although several evidence indicate that cigarette smoking induces systemic oxidative stress in humans (reviewed in [2]), data from *in vitro* [36] and clinical studies [37] have shown that smoking is also associated with a decline in the endogenous GSH-dependent detoxification systems [1], [2]. In the present study, the evidence of increased concentrations of oxPAPC, a reliable marker of oxidative stress [5], and of decreased concentrations of GSH in serum and in PBMC of young heavy smokers is in line with our previous data showing a progressive increase of oxidative stress in PBMC of young smokers (from moderate to heavy), which was coupled with a progressive decrease of GSH [21].

FMD of the brachial artery is currently the most frequently used non-invasive surrogate of endothelial function [22], [23], and it has been convincingly demonstrated to reflect endothelium-dependent vasodilation mediated by NO [38]. In this study we found that FMD was impaired in young heavy smokers, whereas GTN-induced vasodilation was not affected. Our results are consistent with previous observations showing a loss of FMD in young smokers [15]–[17], without affecting smooth muscle cell function [17].

Although it has been suggested that cigarette smoking produces endothelial dysfunction mainly by reducing NO bioavailability [15], [18], [19], the precise mechanism is complex and remains under investigation. The majority of *in vitro* data has involved the use of CS extract (CSE); however, this solution is probably not physiological, because in the circulation, certain toxic components may be neutralized by the antioxidants present in the blood (15). Therefore to simulate a physiological environment, serum from the subjects was used as a vehicle of CS exposure in our *in vitro* model. In the present report we provide evidence that smokers' serum increased ROS formation and reduced NO production in HUVECs, compared to serum from the non-smokers' group. Under similar experimental conditions, these results are consistent with earlier demonstrations that endothelial cells exposed to smokers' serum decreased NO production and endothelial NO synthase activity [15], [39].

To clarify if this reduced NO production was due to the higher concentrations of oxPAPC found in smokers' serum, and based on our recent data that PGPC dose-dependently increased the generation of ROS in PBMC [21], we exposed HUVECs to increasing concentrations of oxPAPC. Our data demonstrated a dose-dependent increase in ROS formation that was paralleled to a dose-dependent basal and bradykinin-stimulated NO decrease in HUVECs. Taken together, these findings indicate that the increased oxidative stress shown in young smokers plays a causal role in the reduced endothelial NO production.

However, our results uniquely demonstrate that FMD was closely associated with the concentrations of GSH in PBMC in all subjects. Although recent data suggest a relationship between decreased plasma GSH concentrations and impairment of postprandial endothelial function in menopausal women [40] and a positive relationship between FMD and plasma GSH in smokers [20], to our knowledge this is the first report that indicates that cellular GSH concentrations correlate with FMD in young subjects. These data, together with the demonstration of decreased levels of GSH in plasma and in PBMC of smokers, led us to speculate that a further important consequence of the increased oxidative stress found in young smokers may be an endothelial GSH depletion. The results from our *in vitro* study confirmed this hypothesis; in fact, smokers' serum compared to non-smokers' serum reduced intracellular GSH concentrations in HUVECs and decreased the expression of Nrf2, of HO-1 and of GCLC, which is the catalytic subunit of the rate-limiting enzyme for the GSH *de novo* synthesis [9]. These results are of particular relevance because it is well known that GSH plays a major role in antioxidant cell protection [9], and that the Nrf2/ARE pathway is crucial for the regulation of intracellular redox state [10].

Recent studies have shown a decline of Nrf2 expression in pulmonary macrophages of current smokers [41] and an increased susceptibility to CS-induced emphysema in Nrf2-deficient mice [42] suggesting a protective role of Nrf2 in CS-lung injury [43]. Moreover we have recently shown that the protective Nrf2/ARE pathway in PBMC was overexpressed only in moderate smokers, whereas it was repressed in heavy smokers [21] indicating that heavy smokers, contrary to moderate smokers, do not appropriately react to the intracellular oxidative stress in terms of Nrf2/ARE activation. Interestingly, in the present study this inadequacy of response was observed not only *in vivo* but also *in vitro*, where we showed that low concentrations of oxPAPC slightly increased GSH and Nrf2/ARE gene expression, whereas oxPAPC at concentration similar to that found in the smokers' serum significantly reduced GSH concentrations and did not cause the activation of the Nrf2/ARE pathway. Our data are in line with recent studies (reviewed in [6]) demonstrating that oxPAPC induces both a complexity of pro-inflammatory and pro-atherosclerotic effects (including increase in intracellular ROS levels and GSH depletion in endothelial cells), as well as tissue-protective and anti-inflammatory activities through induction of phase II genes mediating protection from oxidant stress. In this context it has been recently shown that low concentrations of oxPAPC (25–75 µg/mL) do not damage cells but induce antioxidant enzymes such as GCLM and HO-1, through activation of Nrf2 [44].

In these conditions, of course, the reduction of GSH may also be the result of a net loss of GSH, since cigarette smoke-dependent ROS have already been shown to cause a fall of reduced GSH not accompanied by an increase of oxidized GSH [45].

Moreover the induction of GCLC and HO-1 appeared to be critically dependent on Nrf2, because suppression of Nrf2 expression by siRNA was associated to a significant reduction of HO-1 and GCLC expression induced by oxPAPC in HUVECs. These data agree with a recent *in vitro* study showing that long term CS exposure led to decreased HO-1 expression concomitantly with nuclear Nrf2 decrease in human macrophage cell line [46]. Furthermore, since recent evidence suggests that HO-1 can improve vascular function by enhancing NO bioavailability [47], the serum oxidative stress-induced repression of HO-1 showed in this study may, at least in part, have contributed to the decreased endothelial NO production.

There is also evidence suggesting a complex and interesting interaction between NO and GSH [48] and in particular a recent study showed that NO increased the synthesis of GSH in endothelial cells by inducing the expression of GCLC and through the activation of Nrf2 [49]. We can therefore speculate that the endothelial NO decrease induced by serum from smokers found in this study may have an additional effect on oxidative stress-induced repression of Nrf2/ARE pathway.

In conclusion, the results of this study support the notion that in young healthy smokers with endothelial dysfunction, a novel further consequence of increased oxidative stress is an endothelial decrease of Nrf2 and GCLC expression with consequent GSH depletion.

Although our *in vitro* results and the correlation between intracellular GSH and FMD suggest that intracellular GSH depletion may have a role in endothelial dysfunction of young smokers, we cannot draw a definite conclusion and further studies are needed.
